# Supplementary material for: Asynchronous magnetic resonance elastography: Shear wave speed reconstruction using noise correlation of incoherent waves
Source: Magn Reson Med. 2022 Oct 27;89(3):990–1001. doi: 10.1002/mrm.29502 (PMC9792433; doi:10.1002/mrm.29502)
Supplement: Supplementary file 1 — DATA S1 MATLAB code used to generate the numerical simulations of Figure 1 [file MRM-89-990-s002.zip › k-Wave/helpfiles/writeFlags.html]

writeFlags :: Functions (k-Wave)


# writeFlags

Write input flags to a k-Wave HDF5 file.

## Syntax

```
writeFlags(filename)
```

## Description

`writeFlags` reads the input HDF5 file and derives and writes the required source and medium flags based on the datasets present in the file. For example, if the file contains a data set named `'BonA'`, the `nonlinear_flag` will be written as true. Conditional flags are also written. The source mode flags are written when appropriate if they are not already present in the file. The default source mode is `'additive'`.

List of flags that are always written

- `ux_source_flag`
- `uy_source_flag`
- `uz_source_flag`
- `p_source_flag`
- `p0_source_flag`
- `transducer_source_flag`
- `nonuniform_grid_flag`
- `nonlinear_flag`
- `absorbing_flag`
- `sensor_mask_type`

List of conditional flags

- `u_source_mode`
- `u_source_many`
- `p_source_mode`
- `p_source_many`

## Inputs

|  |  |
| --- | --- |
| `filename` | name of HDF5 file to write matrix to |

## See Also

`h5writeatt`, `writeAttributes`, `writeGrid`, `writeMatrix`
